# Diagnostic value of procalcitonin and presepsin for sepsis in critically ill adult patients: a systematic review and meta-analysis

**DOI:** 10.1186/s40560-019-0374-4

**Published:** 2019-04-15

**Authors:** Yutaka Kondo, Yutaka Umemura, Kei Hayashida, Yoshitaka Hara, Morio Aihara, Kazuma Yamakawa

**Affiliations:** 1 Department of Emergency and Critical Care Medicine, Juntendo University Urayasu Hospital, 2-1-1 Tomioka, Urayasu, Chiba 279-0021 Japan; 2 Department of Traumatology and Acute Critical Medicine, Osaka University Graduate School of Medicine, 2-15 Yamadaoka, Suita, Osaka 565-0871 Japan; 3 Department of Emergency and Critical Care Medicine, Keio University School of Medicine, 35 Shinanomachi, Shinjuku-ku, Tokyo, 160-8582 Japan; 4 Department of Anesthesiology and Critical Care Medicine, Fujita Health University School of Medicine, 1-98 Dengakugakubo, Kutsukakecho, Toyoake, Aichi 470-1192 Japan; 5 Department of Gastroenterology and Hematology, Hirosaki University Graduate School of Medicine, 1-bunkyocho, Hirosaki, Aomori 036-8560 Japan; 6 Division of Trauma and Surgical Critical Care, Osaka General Medical Center, 3-1-56 Bandai-Higashi, Sumiyoshi-ku, Osaka, 558-8558 Japan

**Keywords:** Sepsis, Procalcitonin, Presepsin, Diagnostic test accuracy, Systematic review, Meta-analysis

## Abstract

**Background:**

Early and accurate diagnosis of sepsis is challenging. Although procalcitonin and presepsin have been identified as potential biomarkers to differentiate between sepsis and other non-infectious causes of systemic inflammation, the diagnostic accuracy of these biomarkers remains controversial. Herein, we performed a comprehensive meta-analysis to assess the overall diagnostic value of procalcitonin and presepsin for the diagnosis of sepsis.

**Methods:**

We searched three electronic databases (MEDLINE, EMBASE, and the Cochrane Central Register of Controlled Trials) for relevant studies. Two authors independently screened articles on the basis of inclusion and exclusion criteria. The pooled sensitivity, specificity, and summary receiver operating characteristic curves were estimated. The quality of evidence for diagnostic accuracy in absolute effects, i.e., the number of true or false positives and true or false negatives, gave a particular pre-test probability.

**Results:**

We included 19 studies (19 observational studies and no randomized controlled trials) that had enrolled 3012 patients. Analyses of summary receiver operating characteristic curves revealed areas under the receiver operating characteristic curves of 0.84 for procalcitonin and 0.87 for presepsin. The pooled sensitivities and specificities were 0.80 (95% confidence interval 0.75 to 0.84) and 0.75 (95% confidence interval 0.67 to 0.81) for procalcitonin. For presepsin, these values were 0.84 (95% confidence interval 0.80 to 0.88) and 0.73 (95% confidence interval 0.61 to 0.82), respectively. There were no statistically significant differences in both pooled sensitivities (*p* = 0.48) and specificities (*p* = 0.57) between procalcitonin and presepsin.

**Conclusion:**

Our meta-analysis provided evidence that the diagnostic accuracy of procalcitonin and presepsin in detecting infection was similar and that both are useful for early diagnosis of sepsis and subsequent reduction of mortality in critically ill adult patients.

**Systematic review registration:**

The study was registered in PROSPERO under the registration number CRD42016035784.

**Electronic supplementary material:**

The online version of this article (10.1186/s40560-019-0374-4) contains supplementary material, which is available to authorized users.

## Background

Sepsis is defined as a life-threatening organ dysfunction caused by a dysregulated host response to infection [1]. Despite recent developments in the management of sepsis patients, morbidity and mortality still remain high [2]. Presently, clinical findings, biological markers, and microorganism isolation comprise the basis for diagnosing sepsis. Recent guidelines emphasize that early diagnosis and timely administration of antimicrobial therapy are crucial in reducing morbidity and mortality in sepsis patients [3]. However, no single clinical or biological marker indicative of sepsis has been adopted unanimously.

Procalcitonin (PCT) is the inactive propeptide of calcitonin, which is released by C cells of the thyroid gland, hepatocytes, and peripheral monocytes. PCT is widely reported as a useful biochemical marker to differentiate sepsis from other non-infectious causes of systemic inflammation. However, recent evidence has yielded conflicting results [4–6], which is reflected by the weak recommendation and the low quality of evidence in the 2016 Surviving Sepsis Campaign (SSC) guideline [3].

Presepsin (P-SEP), the newly identified infection biomarker, is a 13 kDa fragment of the N-terminal of soluble CD14 and is released into the blood upon the activation of monocytes in response to infection [7–10]. Although P-SEP appeared to be comparable to other inflammatory biomarkers, i.e., C-reactive protein, interleukin-6, and PCT, in the diagnosis of sepsis [11], there has been limited meta-analytical evidence on the diagnostic performance of P-SEP with PCT.

We thus sought to summarize the current clinical evidence regarding the diagnostic test accuracy (DTA) for PCT and P-SEP and analyze the diagnostic performance of both biomarkers in distinguishing sepsis from non-infectious inflammation in the critical care setting more comprehensively.

## Methods

### Protocol registration

This study complied with the recommendations for the conduct and reporting of systematic reviews and meta-analyses, set forth by the Preferred Reporting Items for Systematic Reviews and Meta-Analyses (PRISMA) statement [12–14], the Meta-Analysis of Observational Studies in Epidemiology proposal [15], and the Cochrane Diagnostic Test Accuracy Working Group [16]. We developed a protocol before conducting the analysis and registered it in PROSPERO (an international prospective register of systematic reviews [http://www.crd.york.ac.uk/PROSPERO/; Registration No. CRD42016035784]). The protocol for this study has been published previously [17].

### Focused review questions

Primary objective: To determine the accuracy of PCT and P-SEP in diagnosing bacterial infection in critically ill adult patients.

Secondary objective: To determine which marker is superior in the diagnosis of bacterial infection in critically ill adult patients.

### Search strategy

We searched the following databases for relevant studies: MEDLINE (via PubMed), EMBASE, and the Cochrane Central Register of Controlled Trials. We developed a search strategy using the combination of keywords and Medical Subject Heading (MeSH)/EMTREE terms, which were “(procalcitonin OR PCT OR presepsin OR “soluble CD14 subtype” OR “sCD14-ST” OR P-SEP) AND (sepsis OR “bacterial infection” OR “systemic inflammatory response syndrome” OR SIRS).” Searches of gray literature and bibliographies of relevant papers were used to complement the results of the search strategies. We did not apply any language restriction to the electronic searches. We also contacted the authors of ongoing or unpublished trials to obtain additional details and information on these trials.

### Inclusion and exclusion criteria

Two investigators (YK and YH) conducted the study selection independently. Any disagreement was resolved by discussion and the participation of a third author (KY), when necessary. We included cross-sectional studies, cohort studies, case-control studies, and randomized controlled trials that evaluated the accuracy of PCT or P-SEP in plasma or serum (index test), when used to diagnose bacterial infection or sepsis in critically ill adult patients (reference standards). In this study, “sepsis” meant “life-threatening organ dysfunction caused by a dysregulated host response to infection,” according to the new definition proposed in 2016 (Sepsis-3) [1]. We also accepted various comparable definitions, such as the severe sepsis/septic shock, with the conventional definition (Sepsis-1, 2) [18]. We excluded the following studies with insufficient information when building a 2 × 2 contingency table: abstracts with inadequate information to enable the assessment of methodological quality and duplicates or sub-cohorts of already published cohorts. We also excluded all studies investigating animals; those predominantly comprising neonates or post-cardiac surgical, heart failure, or perioperative patients; and those comprising healthy participants as controls.

### Data extraction and synthesis

The characteristics of all included studies were extracted by two authors (YK and YH). We used 2 × 2 tables to cross-tabulate the positive or negative numerical data from the index test results (positive or negative) against the target disorder. We displayed all the results in various tables. To visually assess the between-study variability, we presented the results in a forest plot, as well as with summary receiver operating characteristic curves (sROC) with 95% confidence interval (CI) using the MIDAS module in STATA software, V.14.0 (Stata Corporation, College Station, Texas, USA). Furthermore, we generated a Fagan’s nomogram, which is a user-friendly graphical depiction of the positive/negative likelihood ratio (LR) by prevalence. Statistical heterogeneity was evaluated informally from the forest plots of the studies’ estimates and more formally using the *χ*^2^ test (*p* < 0.1, significant) and *I*^2^ statistic (*I*^2^ > 50% = significant) with 95% CI.

We conducted sensitivity analyses to determine the robustness of the meta-analyses and to explore the sources of potential heterogeneity in sensitivity and specificity. We performed univariate meta-regression analysis using the following as covariates with 95% CI: risk of bias, year of publication (after 2016 or before 2015), prevalence (< 50% or ≥ 50%), sample size (< 100 or ≥ 100), setting (inside ICU or outside ICU), comorbidities (whether the studies excluded patients who had comorbidities that were likely to influence P-SEP levels), clinical diagnostic criteria (Sepsis-1, 2, or Sepsis-3), causal pathogens of infection (bacteria only or mixed with fungal, viral, or other pathogens), and the cutoff values for each biomarker (< 1.0 or ≥ 1.0 for PCT, < 500 or ≥ 500 for P-SEP).

### Assessment of risk of bias

The qualities of the included studies were independently assessed by two authors (YK and YU) and verified by a third author (KY), when necessary. The study quality of each article was reported using the Quality Assessment of Diagnostic Accuracy Studies (QUADAS-2) tool [19]. We specifically assessed the presence of spectrum, threshold, disease progression, and partial or differential verification bias.

### Rating of the quality of effect estimates

We applied the Grading of Recommendations Assessment, Development, and Evaluation (GRADE) approach to rate the quality of the evidence [20]. The quality of evidence, which reflects the extent to which we are confident that an estimate of the effect is correct, is rated for each outcome across studies (i.e., for a body of evidence). Although the quality of evidence represents a continuum, the GRADE approach provides the rating for the quality of the body of evidence in one of the four grades: high, moderate, low, or very low, defined as follows:

- High: We are very confident that the true effect lies close to that of the effect estimate.
- Moderate: We are moderately confident of the effect estimate and that the true effect is likely to be close to the effect estimate, but there is a possibility that it is substantially different.
- Low: Our confidence in the effect estimate is limited, such that the true effect may be substantially different from the effect estimate.
- Very low: We have very little confidence in the effect estimate, such that the true effect is likely to be substantially different from the estimate of effect.

Confidence ratings may decrease when there is increased risk of bias, inconsistency, imprecision, indirectness, or concern about publication bias.

In contrast to the therapeutic intervention, the GRADE approach suggests different criteria when the evidence comes from studies on diagnostic accuracy. Valid diagnostic accuracy studies—cross-sectional or cohort studies, in patients with diagnostic uncertainty, and in direct comparison of test results with an appropriate reference standard—provide high-quality evidence. However, they are often downgraded to lower quality evidence, because they are liable to limitations, particularly indirectness of outcomes, i.e., uncertainty about the link between the test accuracy and outcomes that are important to patients.

For each outcome, the quality of evidence was started on a high grade, became downgraded by one level when there was a serious issue identified, and by two levels when there was a very serious issue identified in each of the factors used in judging the quality of evidence.

### Importance of DTA outcomes

In DTA, the importance of outcomes, including absolute effects (true positive, true negative, false positive, or false negative), was ranked according to their importance in decision-making as follows: critical, important, or of limited importance. We ranked all four outcomes as critical because both accurate diagnosis and misdiagnosis could influence mortality in critically ill patients.

## Results

### Results of the search

**Fig. 1:**
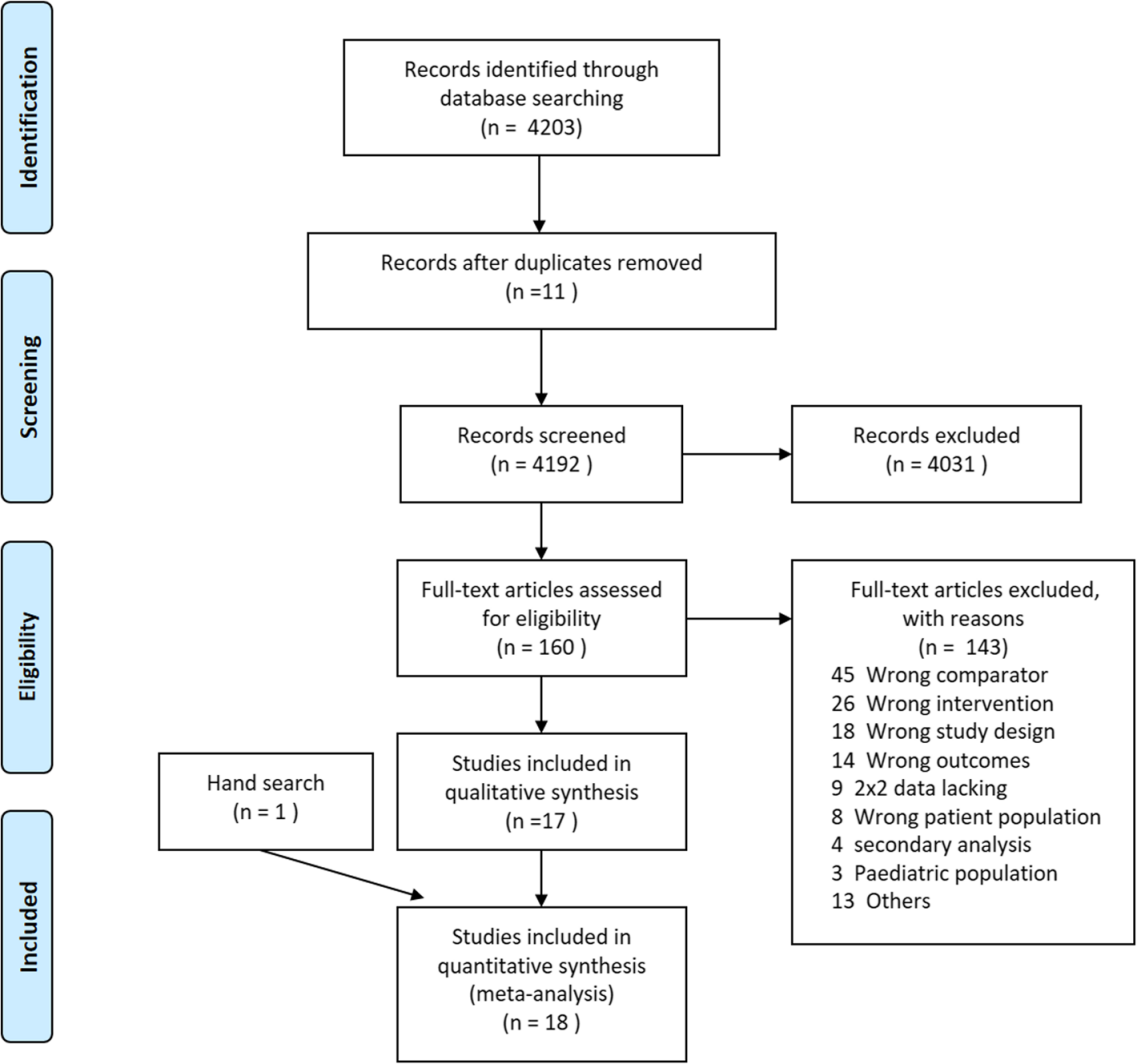
Flow chart of the identification and selection of studies for inclusion

We identified 4203 potentially eligible articles at the initial search (Fig. 1), of which 4192 articles were retained after de-duplicating. After screening the titles and abstracts, 4031 articles were found to be clearly irrelevant and were excluded. We retrieved the full texts of the remaining 160 records (155 observational studies and 5 randomized controlled trials) and assessed them for eligibility. Finally, we included 19 studies (19 observational studies and no randomized controlled trials) that enrolled 3012 patients [11, 21–37]. Among these, 18 [11, 21–23, 25–37] studies evaluated the diagnostic values of PCT in infection and 10 [11, 21, 24–26, 28–30, 33] determined the diagnostic accuracy of P-SEP in infection.

### Basic features of included studies

The characteristics of the included studies are summarized in Table 1. The earliest article [34] was published in 1999 while 18 were published after 2000, with 15 [11, 21, 23–26, 28–31, 33, 35–37] being published after 2010. Twelve studies [24, 26–32, 34–36] took place in Europe, 5 in Asia [11, 22, 25, 33, 37], and 1 each in North America [23] and Africa [21]. Seventeen [11, 21–27, 29, 30, 32–37] studies were conducted prospectively, and the others were retrospective. All studies described diagnostic cutoff thresholds for PCT or P-SEP. The cutoff thresholds widely varied between > 0.28 and > 4.5 ng/ml for PCT and between > 101.6 and > 1000 pg/ml for P-SEP.

**Table 1:** Characteristics of the included studies

Author, year	Country	Number of participants	Mean/median age	Study design	Sepsis definition	Cutoff value		Prevalence	Sensitivity		Specificity	
						PCT (ng/ml)	P-SEP (pg/ml)		PCT	P-SEP	PCT	P-SEP
Ali, 2016 [21]	Egypt	51	49.8	Prospective	Sepsis-3	0.85	907	0.647	60.6%	69.7%	88.9%	83.3%
Balci, 2003 [22]	Turkey	89	58	Prospective	Sepsis-1	2.415	–	0.461	85.4%	–	91.7%	–
Bauer, 2016 [23]	USA	219	59	Prospective	Sepsis-1	0.74	–	0.551	73.1%	–	74.2%	–
Behnes, 2014 [24]	Germany	116	*62*	Prospective	Sepsis-2	–	530	0.705	–	91.0%	–	53.6%
ÇakirMadenci, 2014 [25]	Turkey	37	40	Prospective	ABA 2007^a)^	0.759	542	0.393	75.4%	77.5%	78.7%	76.5%
Endo 2012 [11]	Japan	185	66	Prospective	Sepsis-2	0.5	600	0.622	86.1%	87.8%	78.6%	81.4%
Enguix-Armada 2016 [26]	Spain	388	63	Prospective	Sepsis-2	0.28	101.6	0.634	92.3%	81.7%	96.5%	96.5%
Gibot 2004 [27]	France	76	60	Prospective	Sepsis-1	0.6	–	0.618	83.0%	–	69.0%	–
Godnic 2015 [28]	Slovenia	47	N.A.	Retrospective	Sepsis-2	3.12	413	0.851	57.5%	85.0%	71.4%	57.1%
Klouche 2016 [29]	France	144	58	Prospective	Sepsis-1	0.5	466	0.694	80.0%	90.0%	59.1%	54.5%
Leli 2016 [30]	Italy	92	73	Prospective	Sepsis-1	4.4	843.5	0.281	84.0%	88.0%	84.4%	71.9%
Miglietta 2015 [31]	Italy	145	64.4	Retrospective	Sepsis-1	0.88	–	0.625	85.7%	–	83.3%	–
Romualdo 2014	Spain	226	67	Prospective	Original	0.45	729	0.164	75.7%	81.1%	64.0%	63.0%
Selberg 2000 [32]	Germany	33	47.9	Prospective	Sepsis-1	3.3	–	0.667	86.4%	–	54.5%	–
Takahashi 2016 [33]	Japan	103	68	Prospective	Sepsis-1	0.85	658	0.85	78.8%	72.9%	73.3%	60.0%
Ugarte 1999 [34]	Belgium	190	62	Prospective	Sepsis-1	0.6	–	0.584	67.6%	–	60.8%	–
vanderGeest 2016 [35]	Netherlands	301	57	Prospective	Original	1.41	–	0.505	65.1%	–	66.4%	–
Wong 2013 [36]	France	270	61	Prospective	Not described	0.5	–	0.537	88.3%	–	64.0%	–
Yang 2016 [37]	China	300	64	Prospective	Sepsis-1	0.4475	–	0.357	83.2%	–	53.9%	–

*PCT* procalcitonin, *P-SEP* presepsin

^a)^American Burn Association Consensus Criteria

### Risk of bias of included studies

**Fig. 2:**
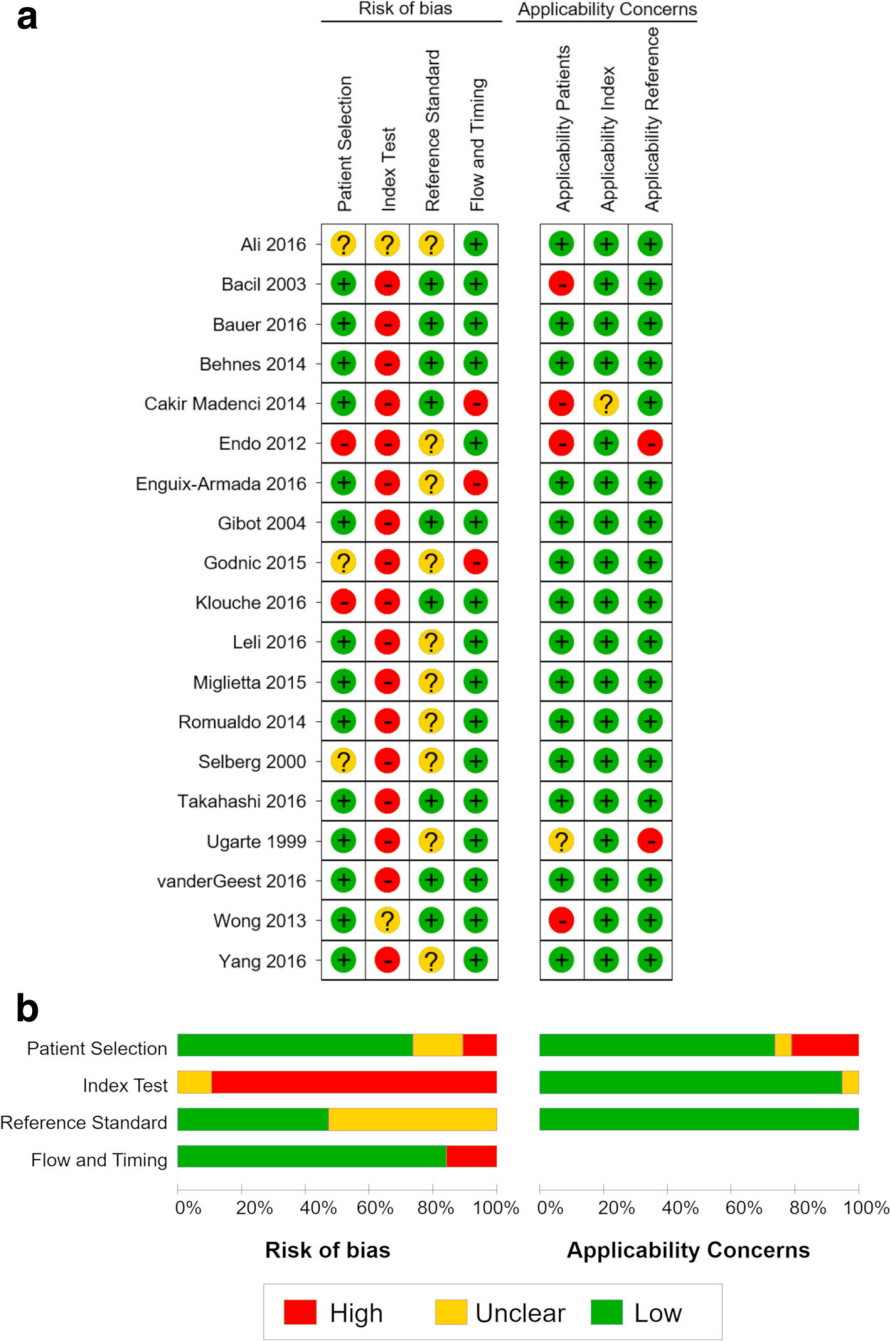
Risk of bias and applicability concerns summary (**a**) and graph (**b**), review authors’ judgements about each domain, for each included study

We illustrated the quality of the included 19 studies using the QUADAS-2 tool (Fig. 2). All studies had unclear or high risk of bias in at least one domain. Five studies [11, 21, 28, 29, 32] demonstrated unclear or high-risk patient selection bias, resulting mainly from inappropriate exclusion criteria and the absence of a clear definition for exclusion criteria. All studies demonstrated unclear or high risk of index test interpretation bias, owing to the lack of a clearly pre-specified cutoff threshold of PCT and P-SEP for a positive diagnosis.

We assigned a high concern for patient selection applicability in four studies [11, 22, 25, 36], which included critically ill adult patients, regardless of suspected bacterial infection. Only one study [25] focusing on severe burn patients was assigned an unclear concern for applicability with respect to the index test, because burns could be a possible source of increased PCT and P-SEP levels. None of the studies had high or unclear concerns for applicability with respect to the reference standard.

### Diagnostic accuracy

**Fig. 3:**
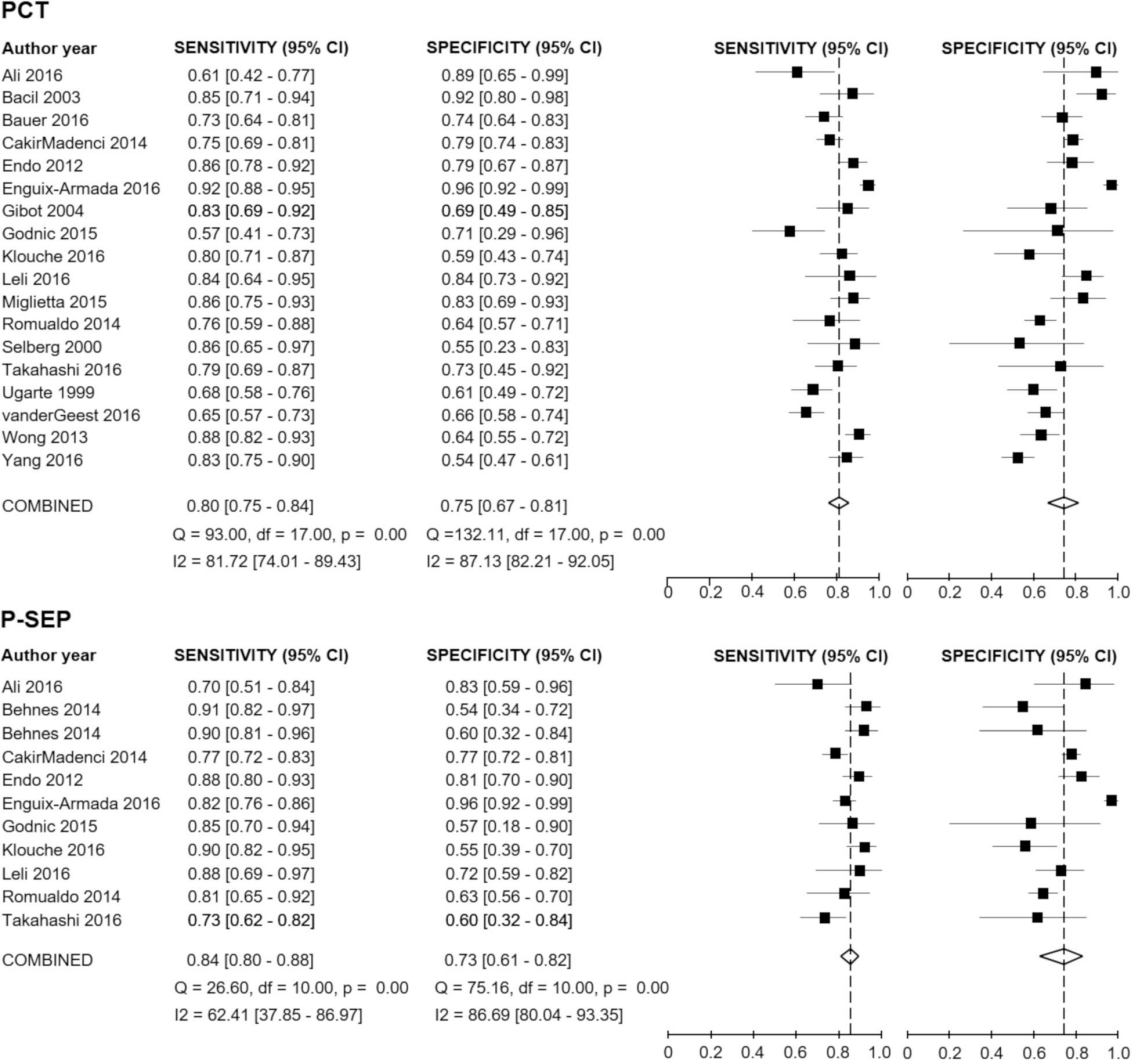
Forest plots of PCT and P-SEP for the diagnosis of infection. The plot shows study-specific estimates of sensitivity and specificity with 95% confidence interval (CI). The studies were ordered according to the study names. PCT, procalcitonin; P-SEP, presepsin

The forest plot in Fig. 3 shows the sensitivity and specificity ranges for PCT and P-SEP for infection, across included studies. The pooled sensitivity for PCT and P-SEP were 0.80 (95% CI 0.75 to 0.84) and 0.84 (95% CI 0.80 to 0.88), respectively. The pooled specificities of these biomarkers were 0.75 for the former (95% CI 0.67 to 0.81, for PCT) and 0.73 for the latter (95% CI 0.61 to 0.82, for P-SEP). Fagan’s nomogram results for PCT indicated that the pooled pre-test probability ratio of 50% by positive/negative LR yielded positive/negative post-test probability ratio of 77% and 22%, respectively. Furthermore, Fagan’s nomogram results for P-SEP demonstrated positive/negative post-test probability ratio of 76% and 19%, respectively. More details can be found in Additional file 1: Figure S1.

**Fig. 4:**
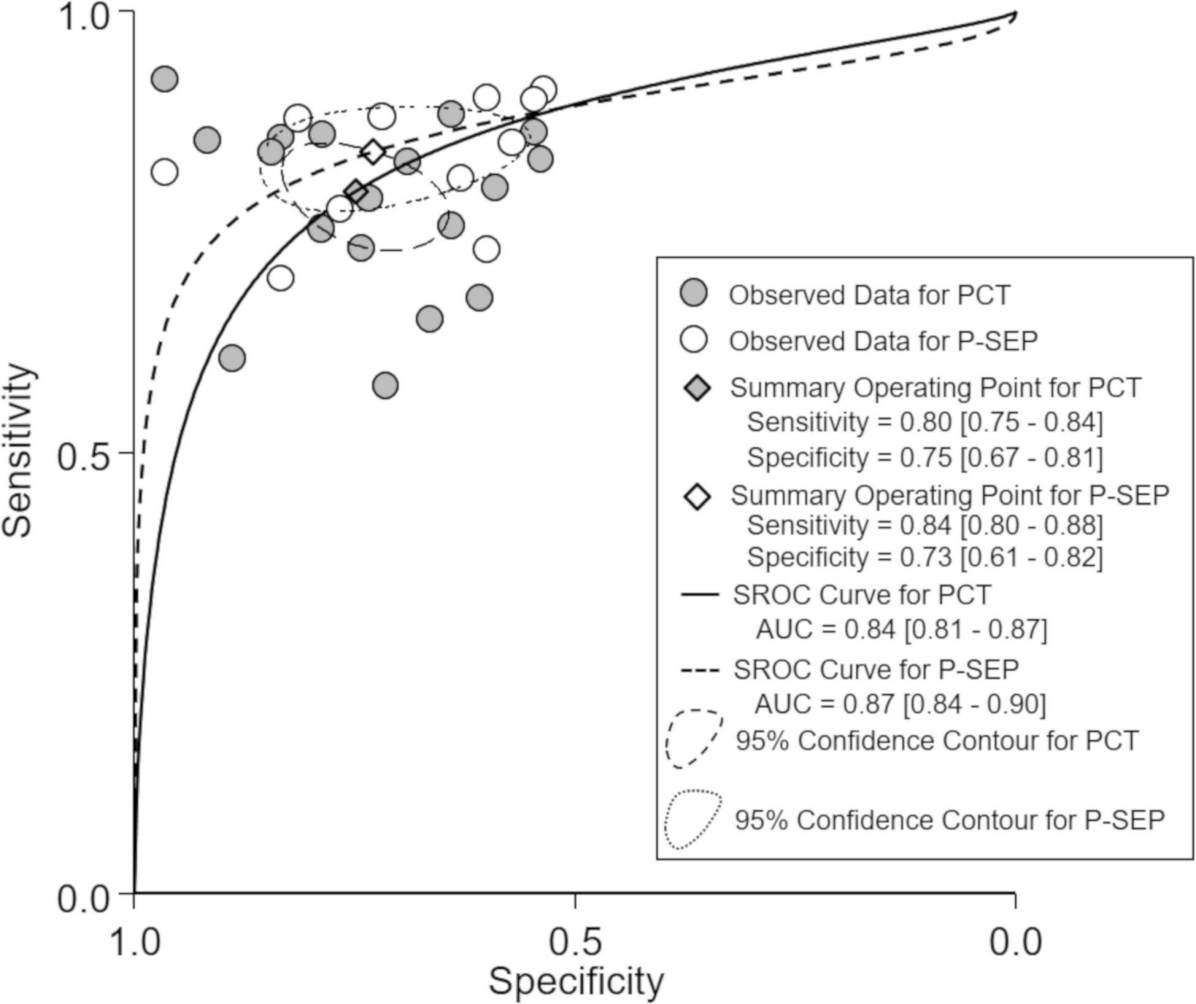
Summary ROC curves of PCT (the solid line) and P-SEP (the dashed line) for the detection of infection. Each pair of points represents the pair of sensitivity and specificity for each evaluation. PCT, procalcitonin; P-SEP, presepsin. The overall diagnostic accuracy of PCT and P-SEP for infection was moderate and comparable

We also constructed the sROC curves and calculated the area under ROC (AUROC) for included studies (Fig. 4). The overall diagnostic performance of PCT and P-SEP for infection were comparable (AUROC 0.84 [95% CI 0.81 to 0.87], and 0.87 [95% CI 0.84 to 0.90], respectively).

### Investigations of heterogeneity

**Fig. 5:**
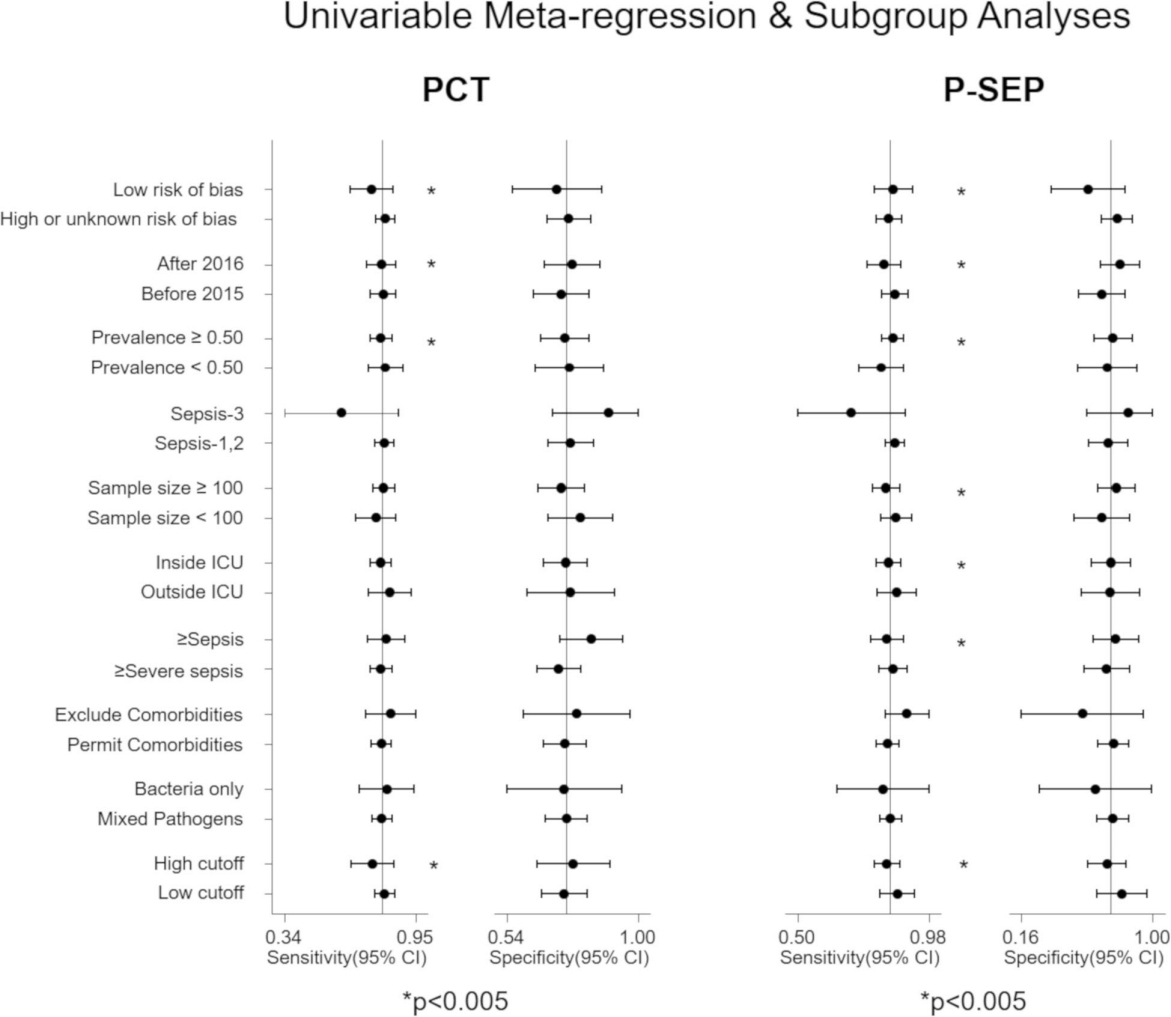
Univariate meta-regression analysis by several possible causes of heterogeneities. PCT, procalcitonin; P-SEP, presepsin. To correct for multiple comparisons with 10 relevant covariates, we considered a *p* value of < 0.05 as statistical significance. The sensitivity of heterogeneity among included studies might be attributable to several factors, such as risk of bias, publication years, and prevalence of infection

Because there were substantial heterogeneities among pooled results of sensitivity and specificity for both PCT and P-SEP (Fig. 3), we performed several sensitivity analyses to explain the heterogeneities by investigating the study characteristics using meta-regression analysis. Univariate meta-regression analysis revealed that the sensitivity of heterogeneity among included studies might be attributable to several relevant factors, such as the risk of bias (low risk or high risk), publication years (until 2015 or after 2015), prevalence of infection (< 50% or ≥ 50%), sample size (< 100 or ≥ 100), study setting (inside ICU or outside ICU), clinical diagnostic criteria (sepsis-1, 2 or sepsis-3), and the cutoff value for each biomarker (< 1.0 or ≥ 1.0 for PCT, < 500 or ≥ 500 for P-SEP) (Fig. 5).

### Head-to-head comparison of the two biomarkers

To compare the diagnostic performance of PCT and P-SEP in similar populations, we evaluated the nine studies which directly compared PCT and P-SEP in the same population [11, 21, 25, 26, 28–30, 33]. As a result, there were no statistically significant differences in both pooled sensitivities (*p* = 0.48) and pooled specificities (*p* = 0.57) between PCT and P-SEP. Besides, we conducted a head-to-head comparison of PCT and P-SEP in several subgroups stratified by study characteristics and found no statistically significant differences between the two biomarkers in any of the subgroups (Additional file 2).

### Publication biases

**Fig. 6:**
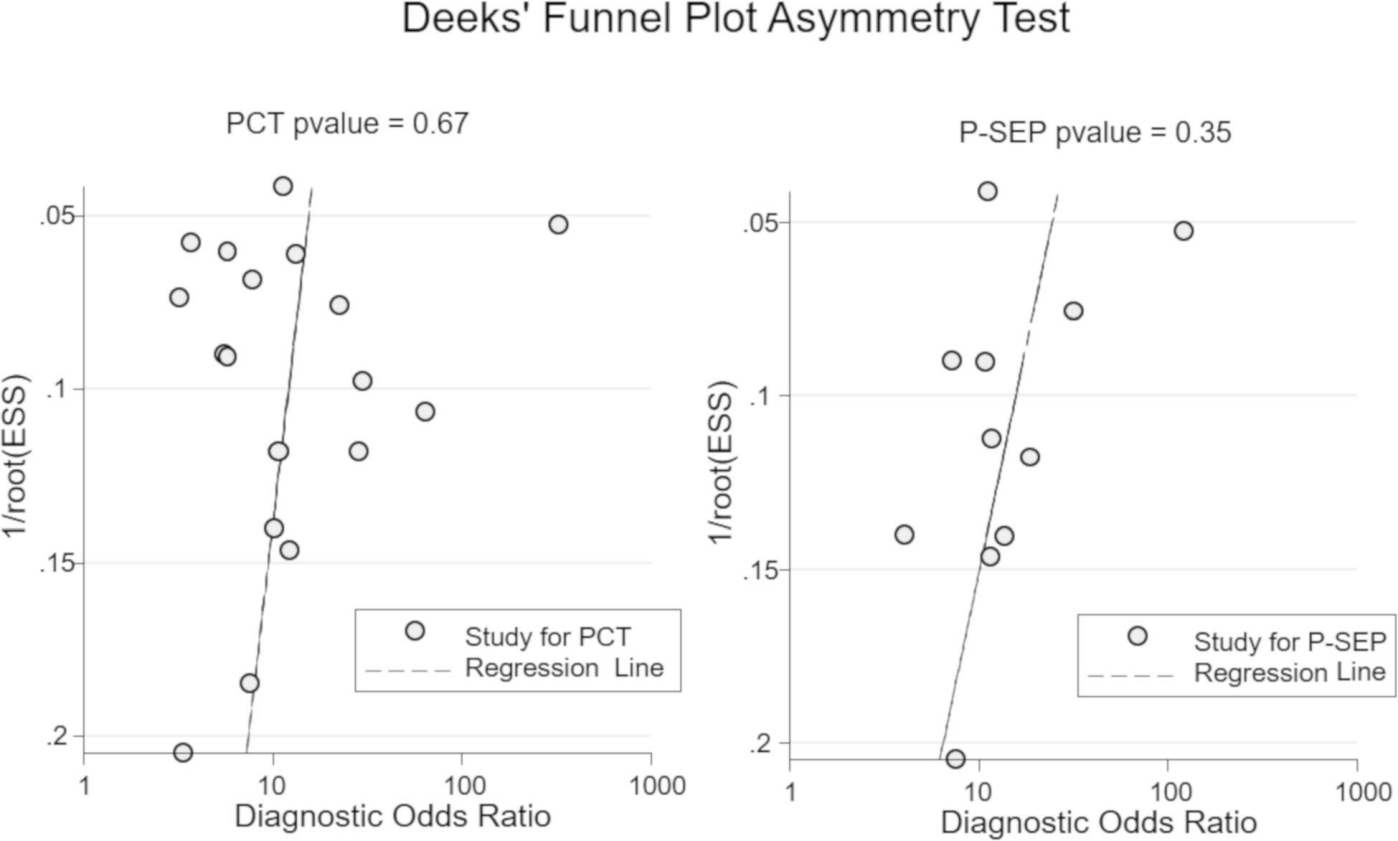
Deeks’ funnel plot to estimate the presence of publication bias. PCT, procalcitonin; P-SEP, presepsin; ESS, effective sample size. We detected no evidence of publication bias (PCT: *p* = 0.67; P-SEP: *p* = 0.35)

We detected no evidence of publication biases, assessed by Deeks’ Funnel Plot Asymmetry Test (PCT: *p* = 0.67, P-SEP: *p* = 0.35) (Fig. 6).

### Quality of DTA evidence using the GRADE system (or approach)

We summarized the main findings and the quality of evidence for each outcome across all the studies in the GRADE evidence profile (Table 2). Because, all included studies showed unclear or high risk of index test interpretation bias, the quality of evidence of the outcomes was downgraded by one level (from high to moderate). Furthermore, with the substantial heterogeneities among pooled estimates of the sensitivity and specificity in included studies, and because the source of heterogeneities could not be fully explained by the results of our sensitivity analysis, the quality of evidence of the outcomes was again downgraded by one level (from moderate to low). We found no serious indirectness and imprecision. Finally, the quality of the body of evidence supporting PCT and P-SEP for the diagnosis of infection were both graded as “low” for true positive, false negative, false positive, and true negative. Consequently, we graded the overall quality of evidence as “low.”

**Table 2:**
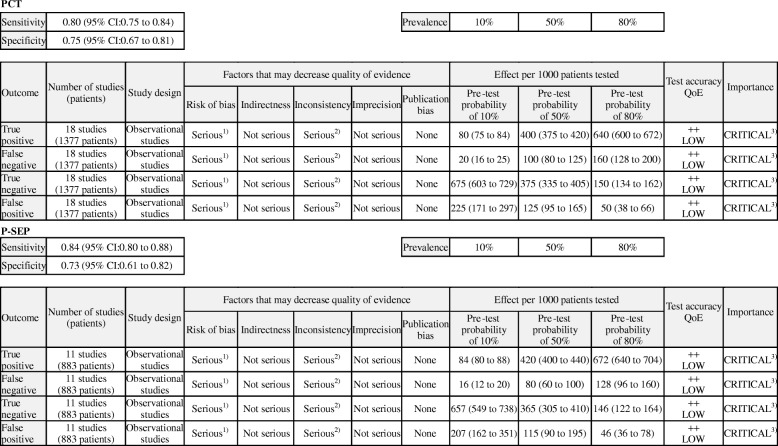
GRADE evidence profile to determine the accuracy of PCT and P-SEP when used to diagnose bacterial infection in adult critically ill patients

The GRADE approach results in an assessment of the quality of a body of evidence in one of four grades: high, moderate, low, or very low. For each outcome, the quality of evidence started at high, were downgraded by one level when there was a serious issue identified, and were downgraded by two levels when there was a very serious issue identified in each factor to judge the quality of evidence

*PCT* procalcitonin, *P-SEP* presepsin, *QoE* quality of evidence

^1)^We downgraded all outcomes for risk of bias because all of the included studies presented an unclear or high risk of index test interpretation bias

^2)^We downgraded all outcomes for inconsistency because there were substantial heterogeneities among pooled results of sensitivity and specificity

^3)^We ranked the importance of all the four outcomes as critical

## Discussion

### Summary of the main results

On the basis of the pre-defined protocol [17], the present meta-analysis and systematic review, which included 19 studies from several regions of the world, assessed the diagnostic accuracy of the index tests in participants with established infection in the critical care setting. To the best of our knowledge, this is the first study using appropriate methodologies and quality assessment tools, which provide evidence that there is no difference in the diagnostic performance of PCT and P-SEP in critically ill patients. Analyses of AUROC revealed AUC values of 0.84 for PCT and 0.87 for P-SEP with modest sensitivity and specificity. Positive and negative LRs for both biomarkers were sufficiently relevant as additional diagnostic tools in cases of infection, which were often indistinguishable from non-infectious disorders in critically ill patients.

Our sensitivity analysis suggested that several factors might have been responsible for the substantial heterogeneity across included studies. There were no obvious threshold effects for both PCT and P-SEP, partly because almost all included studies claimed that in each dataset, the post-specified cutoff threshold calculated by ROC analyses maximizes the diagnostic performance of either biomarker.

### Roles of procalcitonin and presepsin in the diagnosis of sepsis

Given that the SSC guideline emphasizes early diagnosis to improve the clinical outcomes in sepsis [3], developing diagnostic strategies for infection is still required for accurate bedside diagnosis of infections. Although PCT has been widely reported to be an optimal biomarker in the diagnosis of sepsis [38, 39], more recent studies have produced conflicting results [4–6]. P-SEP, which is released into circulation after the activation of the pro-inflammatory signaling cascade upon contact with infectious agents [40], is emerging as a novel circulating marker for sepsis [9]. However, the clinical value of these biomarkers, independently or in combination, is still at investigative stages. Indeed, there are limited meta-analyses and systematic reviews comparing the prognostic performance of P-SEP with PCT for the diagnosis of early-stage sepsis in critically ill patients. Thus, there is a lack of evidence to suggest the relevance of the triaging for these tests. Besides, it is still unclear whether testing for these biomarkers is an addition to the existing tests or a replacement, whether partial or complete.

### Quality of the evidence

The quality of the body of evidence supporting PCT and P-SEP for the diagnosis of infection was judged as “low” for both markers. As almost all included studies did not pre-specify a clear cutoff threshold of PCT and P-SEP for a positive diagnosis (thus indicating an unclear or high risk of index test interpretation bias), we downgraded the quality of evidence by one level for the risk of bias. Consequently, we suggested the measurements of PCT and P-SEP as an optional diagnostic tool for infection and sepsis in critically ill patients. However, further researches are likely to have an important impact on our findings, which may result in changes to the recommendation.

### Strength of the review

Several methodological strengths have enhanced the validity and applicability of our findings. This systematic review and meta-analysis included: (1) any study that measured PCT or P-SEP levels in critically ill adult patients with suspected sepsis, in whom the confirmation of sepsis was by clinical diagnosis or by microbiological confirmation of infection in cultures, or both; (2) comprehensive systemic search without any language restriction to the electronic searches (including 4203 published studies); (3) main results were discussed both by direct comparison and by subgroup analyses; (4) detailed subgroup analyses were performed to solve the heterogeneity concerns; and (5) quality of DTA evidence was rated through a transparent and structured process based on the GRADE approach.

### Agreements and disagreements with other studies

Recently, one meta-analysis comparing P-SEP with other biomarkers (PCT and C-reactive protein) reported that P-SEP has similar diagnostic accuracy as PCT or C-reactive protein [41]. The most important feature distinguishing our analysis from this previous meta-analysis is that we focused only on studies evaluating participants with critical illnesses, such as acute respiratory distress syndrome and sepsis, but not healthy volunteers. The former study had high heterogeneity likely because it included normal healthy volunteers as controls. Thus, it should be interpreted more cautiously. As they reported, methods, to distinguish sepsis from non-infectious causes of inflammation, are necessary in clinical situations [41].

### Limitations of the review

This study has several limitations. First, the microbiological approaches used for the definitive diagnosis of infection varied from one study to another; therefore, some degree of misclassification bias might have existed. Second, most included studies did not report the pre-specified cutoff thresholds for the biomarkers, which might have increased the risk of bias. Finally, since there was insufficient number of data available, we could not compare the bacterial, viral, and fungal infection population groups only. The usefulness of PCT and P-SEP in viral and fungal infection groups remains unknown.

## Conclusion

Our meta-analysis suggests that both PCT and P-SEP are helpful biomarkers for the early diagnosis of sepsis in critically ill adult patients. However, considering the need to avoid misdiagnosis or delayed diagnosis, the use of PCT or P-SEP tests in combination with other clinical modalities for sepsis diagnosis is recommended to improve diagnostic accuracy and patient outcomes.

## Additional files

Additional file 1: **Figure S1.** Fagan’s nomogram of PCT and P-SEP to calculate the positive/negative post-test probabilities of infection. PCT, procalcitonin; P-SEP, presepsin, LR, likelihood ratio. (TIF 2035 kb)

Additional file 2: Direct comparison by univariate meta-regression analysis. Direct comparison by univariate meta-regression analysis. PCT, procalcitonin; P-SEP, presepsin. In any subgroup, we found no statistically significant differences in pooled sensitivities and specificities between PCT and P-SEP. (DOCX 22 kb)

## Abbreviations

AUROC: Area under ROC
CI: Confidence interval
DTA: Diagnostic test accuracy
GRADE: Grading of Recommendations Assessment, Development, and Evaluation
ICU: Intensive care unit
LR: Likelihood ratio
MESH: Medical Subject Heading
PCT: Procalcitonin
PRISMA: Preferred Reporting Items for Systematic Reviews and Meta-Analyses
P-SEP: Presepsin
QUADAS-2: Quality Assessment of Diagnostic Accuracy Studies
ROC: Receiver operating characteristic
SSC: Surviving Sepsis Campaign
