# Supplementary figures and images for: Diagnostic value of procalcitonin and presepsin for sepsis in critically ill adult patients: a systematic review and meta-analysis
Source: J Intensive Care. 2019 Apr 15;7:22. doi: 10.1186/s40560-019-0374-4 (PMC6466719; doi:10.1186/s40560-019-0374-4)

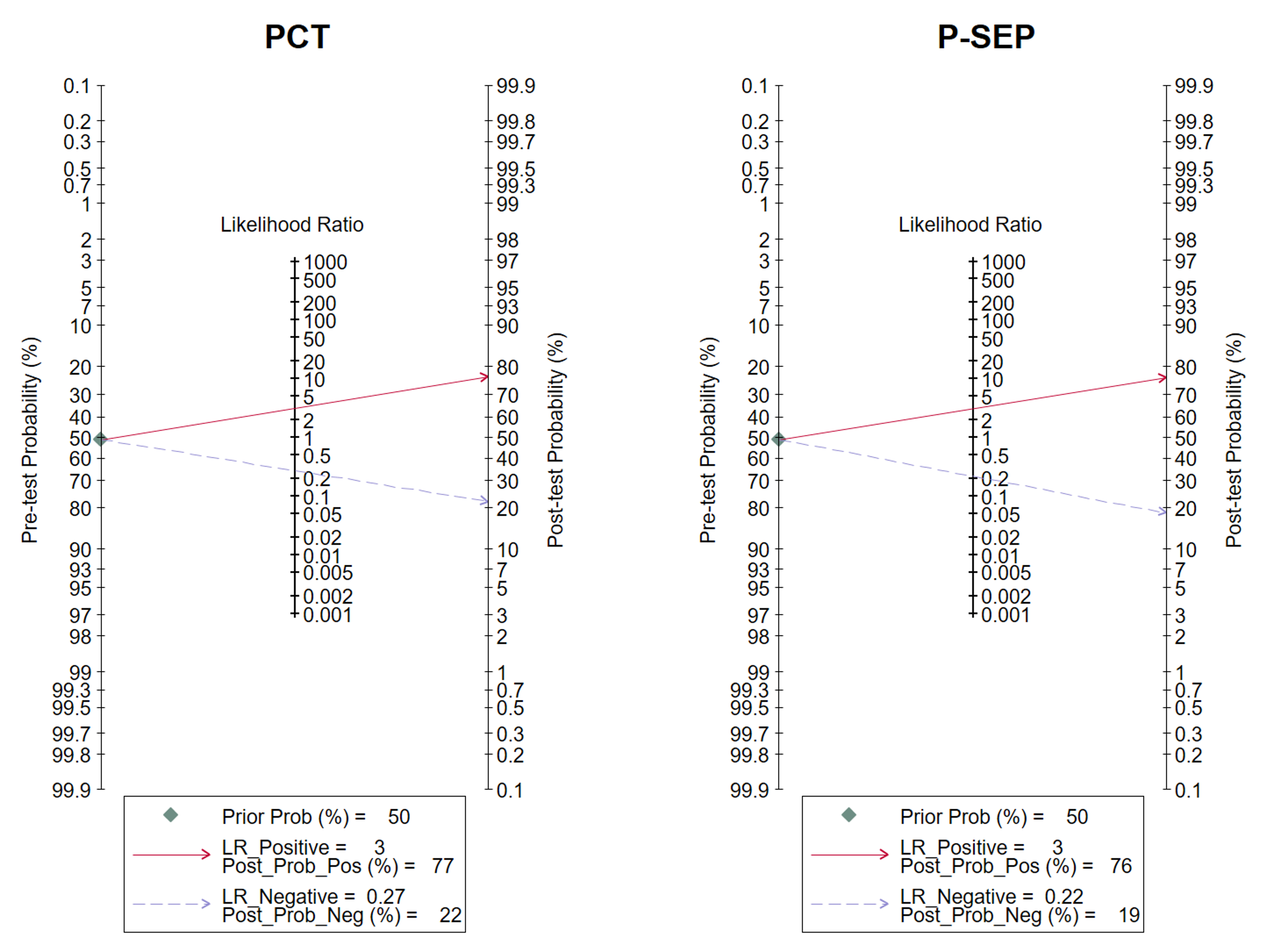

Supplement: Supplementary file 1 — Figure S1. Fagan’s nomogram of PCT and P-SEP to calculate the positive/negative post-test probabilities of infection. PCT, procalcitonin; P-SEP, presepsin, LR, likelihood ratio. (TIF 2035 kb) [file 40560_2019_374_MOESM1_ESM.tif]
