# Supplementary material for: Diagnostic value of procalcitonin and presepsin for sepsis in critically ill adult patients: a systematic review and meta-analysis
Source: J Intensive Care. 2019 Apr 15;7:22. doi: 10.1186/s40560-019-0374-4 (PMC6466719; doi:10.1186/s40560-019-0374-4)
Supplement: Supplementary file 2 — Direct comparison by univariate meta-regression analysis. Direct comparison by univariate meta-regression analysis. PCT, procalcitonin; P-SEP, presepsin. In any subgroup, we found no statistically significant differences in pooled sensitivities and specificities between PCT and P-SEP. (DOCX 22 kb) [file 40560_2019_374_MOESM2_ESM.docx]

Direct comparison by univariate meta-regression analysis. PCT, procalcitonin; P-SEP, presepsin. In any subgroup, we found no statistically significant differences in pooled sensitivities and specificities between PCT and P-SEP.
